# Persistent Postprandial Angina in a Patient With Gastroesophageal Reflux Disease: A Diagnostic Dilemma

**DOI:** 10.7759/cureus.9789

**Published:** 2020-08-16

**Authors:** Joshua K Salabei, Troy J Fishman, Zekarias T Asnake, Matthew Calestino

**Affiliations:** 1 Internal Medicine, University of Central Florida College of Medicine, Hospital Corporation of America North Florida Division, Gainesville, USA; 2 Internal Medicine, North Florida Regional Medical Center, University of Central Florida, Gainesville, USA

**Keywords:** coronary steal, angiography, coronary artery disease, post-prandial pain, angina, pci, case report

## Abstract

Chest pain (CP) is a common reason for visits to the emergency department (ED). The underlying etiology of a good number of cases of CP can be diagnosed with adequate history taking and routine laboratory testing. However, atypical presentations of CP, in the settings of other causes of CP such as gastroesophageal reflux disease (GERD), can sometimes be tricky to diagnose with only routine lab tests and electrocardiogram (EKG). Herein, we present a 73-year-old male with a history of GERD and coronary artery disease who presented to our ED complaining of postprandial CP unaffected by exertion or rest. Initially, his symptoms were thought to be GERD-related but other heart-related causes of CP were considered due to the persistence of his CP postprandially. A cardiac stress test was subsequently done to rule out possible cardiac causes of his CP. His stress test was abnormal prompting heart catheterization that showed almost complete occlusion of his left anterior descending (LAD) and left circumflex (LCx) arteries. His symptoms resolved post-catheterization/stenting of his LAD and LCx arteries. He was later discharged unconditionally. His presentation highlights the required vigilance physicians must maintain when interrogating CP, even when other non-cardiac-related causes seem more plausible.

## Introduction

Chest pain (CP) is a common reason for visits to the emergency department (ED). Multiple etiologies for CP have been described in the literature, and a good number of these etiologies can be diagnosed with adequate history taking and routine laboratory testing [1,2]. However, atypical presentations of CP, in the settings of other causes of CP such as gastroesophageal reflux disease (GERD), can sometimes be tricky to diagnose with only routine evaluations. For example, a patient presenting with postprandial CP unaffected by exertion/rest can be easily misdiagnosed as a non-cardiac CP, especially if initial evaluation involving troponin measurements and electrocardiogram (EKG) are unremarkable. Such misdiagnosis can be fatal; therefore, physicians are cautioned to be on the guard when interrogating CP even when other non-cardiac causes seem more plausible. This case highlights the importance of objective assessment of CP especially in patients with a history of coronary artery disease (CAD).

## Case presentation

**Figure 1:**
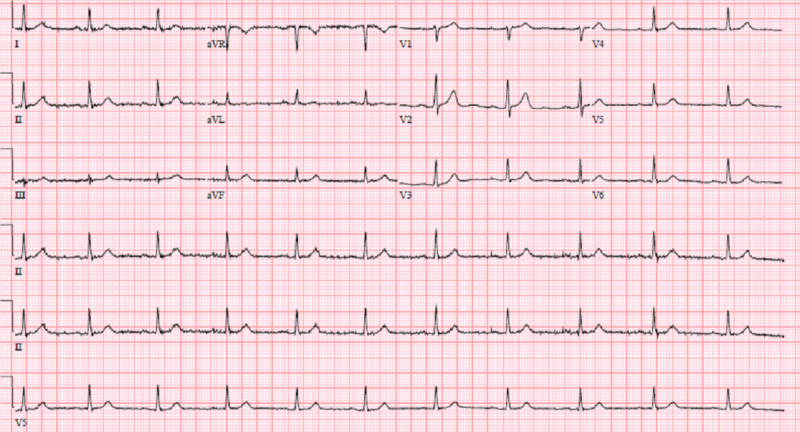
Electrocardiogram at presentation.

We present the case of a 73-year-old man with a past medical history of CAD with a stent in the right coronary artery (RCA) placed seven years earlier and chronic GERD with Barrett’s esophagus, who presented to our ED complaining of a dull non-radiating CP for several weeks. He was lost to follow-up after the placement of his RCA stent. He described his CP as episodic, occurring postprandially, and unaffected by strenuous activity. Pain is not associated with palpitations or diaphoresis. On physical exam, the pain was non-reproducible. The remainder of his physical exam was also non-contributory. Upon presentation to the ED, serial troponins were within normal limits and his EKG showed no ST elevations (Figure 1).

His other laboratory tests are shown in Table 1.

**Table 1:** Laboratory results at the time of presentation WBC: white blood cell count, AST: aspartate aminotransferase, ALT: alanine aminotransferase

Test	Value on Admission	Reference
Hemoglobin	15.2 g/dL	12.0-15.0 g/dL
WBC	9.8 thou/mm^3^	4.5-11.0 thou/mm^3^
Alkaline phosphatase	54 units/L	46-116 units/L
AST	8 units/L	15-37 units/L
ALT	17 units/L	16-61 units/L
Troponins	0.019 ng/mL	<0.05 ng/mL

**Figure 2:**
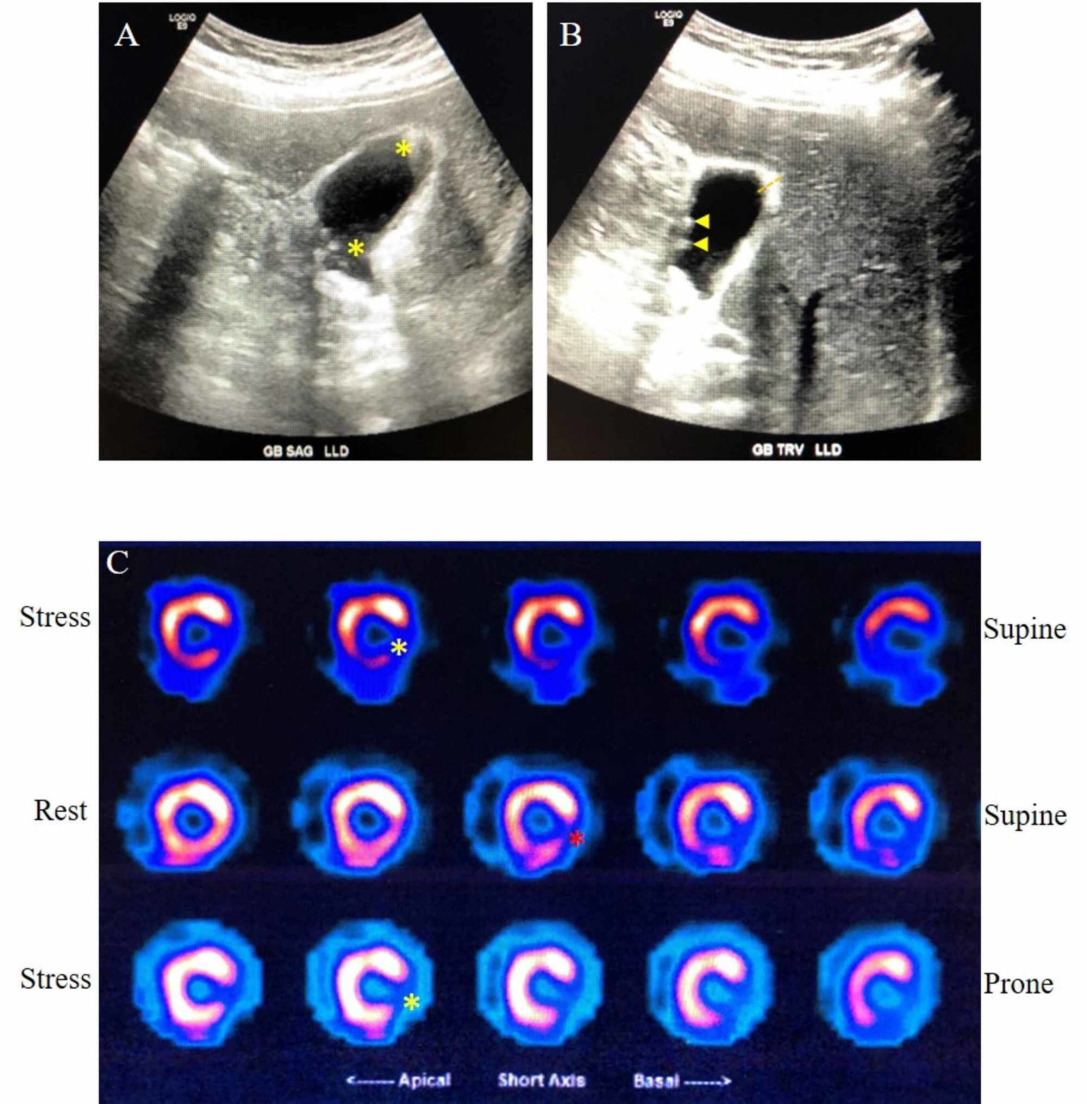
Representative gallbladder and myocardial perfusion scan images (A) Sagittal view of gallbladder (GB) showing sludge (yellow asterisks). (B) Transverse view of GB showing gallstones indicated with yellow arrowheads, and  thickened GB wall indicated with the orange line. (C) Representative myocardial perfusion scan images. Reversible (yellow asterisks) and irreversible changes (red asterisk) after stress are noted. Irreversible areas represent ischemic regions from prior myocardial infarct.

Upper abdominal ultrasound was significant for cholelithiasis with sludge and a thickened gallbladder wall of 4 mm without sonographic Murphy’s sign (Figure 2A, 2B). He was treated with pantoprazole and provided with other supportive measures. Plans for elective cholecystectomy were made. However, he continued to experience postprandial CP while in the hospital, prompting further evaluation. Owing to his history of CAD with a stent and lack of follow-up since first stent placement, suspicion for postprandial angina became high on the differentials. Therefore, an echocardiogram was performed, and it showed an ejection fraction of 55%-60% without wall motion abnormalities. A myocardial perfusion scan (Regadenoson nuclear stress test) showed ischemia in the mid-basal inferolateral wall (Figure 2C).

**Figure 3:**
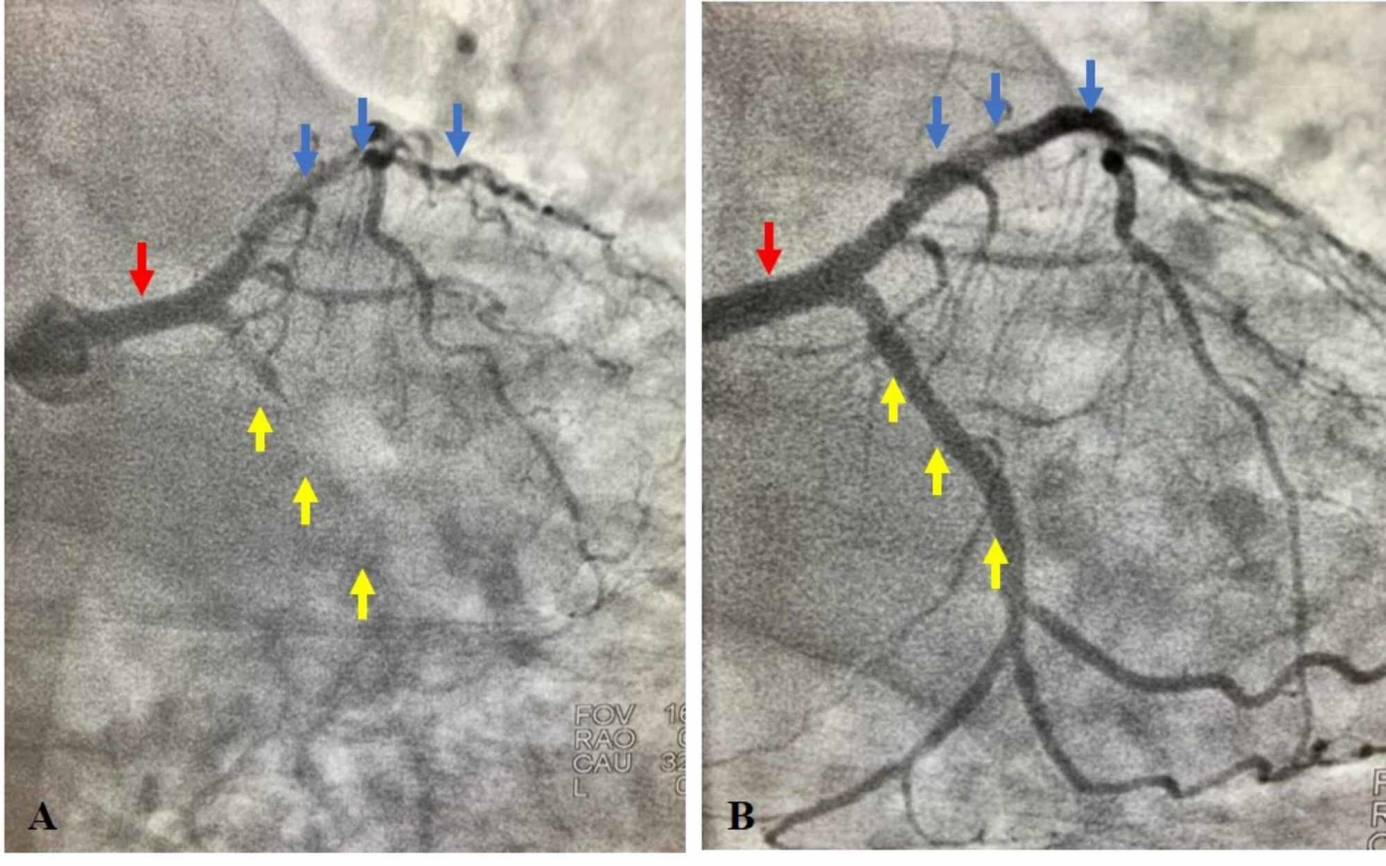
Coronary perfusion scan before and after revascularization (A) Coronary perfusion scan before revascularization. The red arrow shows the LCA with its two main branches: the LCX and the LAD. The yellow arrows indicate the presumed track of the LCX artery. The blue arrows indicate the LAD, showing severe stenosis in its subsequent downstream branches. (B) Post-revascularization. The red arrow shows the LCA with its two main branches; the LCX and the LAD. The yellow arrows indicate the LCX artery, showing restoration of blood flow. The blue arrows indicate the LAD, also showing restoration of blood flow to its downstream branches. LCA; left coronary artery, LAD; left anterior descending artery, LCX; left circumflex artery

Subsequent coronary catheterization revealed severe CAD with 100% occlusion of the left circumflex (LCX) and 90% occlusion of the left anterior descending (LAD) arteries (Figure 3). Both stenosed arteries were successfully revascularized with stents, leading to a complete resolution of his postprandial symptoms. He was then discharged on aspirin and clopidogrel and was advised to follow up with his cardiologist and primary care physician

## Discussion

With CAD among the main causes of death in the USA and worldwide, physicians must recognize subtle and atypical presentations of cardiac-related causes of CP that could be detrimental if missed [3]. Our case highlights this in that (I) the patient had a history of chronic GERD with Barrett’s esophagus, (II) his CP was only exacerbated postprandially, and (III) abdominal ultrasound was significant for cholelithiasis and gallbladder wall thickening. Thus, his history and ultrasound findings, if not interpreted with caution, could serve as potential distractors from the actual cause of his symptoms. Therefore, especially in patients with a history of CAD, any form of CP, typical or atypical, must be interrogated with caution.

The patient had complete occlusion of the LCX and 90% occlusion of the LAD arteries (Figure 2). With this degree of occlusion, it is surprising that his symptoms were not exacerbated by strenuous activity, but rather only postprandially. One possible explanation for this is that the fully patent RCA was sufficient, in addition to collateral circulation, to supply the entire left ventricle.

Over the last couple of years, it has been increasingly recognized that blood supply via redistribution of myocardial blood flow could be the main cause of postprandial angina. For example, contrasting increased myocardial oxygen demand as a mechanism of postprandial angina, a study, using positron emission tomography and high-frequency pulse-wave Doppler echocardiography to measure coronary blood flow in the LAD coronary artery, demonstrated that myocardial blood flow decreases in the territory supplied by stenotic coronary arteries postprandially [2-4]. Still, the exact mechanism by which our patient’s symptoms were exacerbated postprandially remains unanswered and although a postprandial EKG could have helped confirm blood flow compromise to the myocardium, it would have been insufficient to delineate a mechanism. 

The sonographic findings of cholelithiasis and gallbladder wall thickening could have been potential distractors to pursuing a cardiac-related etiology of the patient’s presenting symptom. In addition, he had a chronic history of GERD with Barret’s esophagus, further serving as distractors. His EKG and trended troponins were all unremarkable, and his echocardiographic findings showed no wall abnormalities. Still, he continued to experience postprandial pain despite all the negative findings. The decision to further evaluate the patient was driven principally by objective clinical judgment, highlighting that each case is unique and must be approached as such. We have presented an atypical presentation of postprandial angina and highlight that CP, especially in patients with a known history of CAD, must be approached with caution.

## Conclusions

In this report, we have presented an atypical presentation of CP in a patient who has a known history of GERD. We have highlighted that in patients with a significant history of cardiovascular disease, CP must be interrogated fully, irrespective of the presence of other obvious causes of CP, such as GERD. Since CP is one of the most leading reasons for hospital visits, it is important that physicians be aware of atypical presentations such as presented here. Objective judgment must be applied on a case-by-case basis. 
